# Speech–language therapy educator reflections on the planning and implementation of education and training during the COVID-19 pandemic

**DOI:** 10.4102/sajcd.v69i2.908

**Published:** 2022-09-09

**Authors:** Urisha Naidoo, Penelope S. Flack, Vrinda Rathiram, Legini Moodley, Saira B. Karrim, Nomfundo Buthelezi, Vuledzani Ndanganeni

**Affiliations:** 1 Discipline of Speech-Language Pathology, School of Health Sciences, University of KwaZulu-Natal, Durban, South Africa

**Keywords:** COVID-19, emergency remote teaching and learning, narrative research, South Africa, speech–language pathology, speech–language therapy educators

## Abstract

**Background:**

Universities across the world experienced lockdown and closure of all learning institutions around March 2020 because of the advent of the coronavirus disease 2019 (COVID-19). This lockdown and closure presented challenges to the traditional pedagogical approaches in the health sciences, which typically include both campus-based and clinical site-focused activities involving face-to-face interactions and work integrated learning. The onset of the COVID-19 pandemic resulted in a shift to emergency remote teaching (ERT) and learning.

**Objectives:**

This study aimed to explore speech–language pathology (SLP) educators’ experiences of the planning and implementation of ERT and learning during the COVID-19 pandemic.

**Method:**

A qualitative, descriptive narrative design was adopted to meet the objectives of the study. Seven SLP educators from a single university in South Africa participated in this study by constructing narratives on their experiences. The narratives were analysed using thematic analysis.

**Results:**

Five themes emerged from the data analysis, and these included (1) uncertainty, (2) educator feelings, (3) capacity development, (4) influence of circumstances on teaching, learning and assessment and (5) troubleshooting. Current findings provide insight into the challenges encountered and strategies utilised by educators in planning and implementing ERT and learning.

**Conclusion:**

Beyond the COVID-19 pandemic, most educators believe that a hybrid model would address some concerns identified, such as that of missing face-to-face contact, but that it would still allow for the full exploitation of online activities for teaching, learning and assessment required during clinical training.

## Introduction

Ten days after the first patient was diagnosed with coronavirus disease 2019 (COVID-19) in South Africa, the South African government declared a national state of disaster and on 27 March 2020 placed the country under lockdown (Adams, Seedat, Coutts, & Kater, 2021). As part of measures to control the spread of this novel virus and manage infections, nonpharmaceutical measures were implemented, and these included prohibition of public gatherings, implementation of bans on national and international travel, adherence to regulated social distancing, enforcement of stringent infection control measures, as well as the closure of schools and higher education institutions where large gatherings could serve as super-spreader events (Du Plessis et al., 2022). In South Africa, a national high-level advisory committee was established to provide advice to the Minister of Health and to establish evidence-based policy guidelines (Abdool Karim, 2020).

The South African Minister of Higher Education and Training, Dr Blade Nzimande, in a press briefing expressed the need for academic institutions of higher learning to successfully complete the 2020 academic year. To do this, universities transitioned to emergency online remote teaching (Czerniewicz et al., 2020). The format, timing and method of roll-out varied across institutions; the university where the current study was conducted adopted a trial period of online learning on 18 May 2020 and an official commencement on 01 June 2020 (S. Songca, personal communication, 06 April 2020).

Traditional pedagogical approaches in the health sciences typically include both campus-based and clinical site-focused activities, involving face-to-face interactions and work-integrated learning (Kumar et al., 2021). However, with the onset of the COVID-19 pandemic and the temporary halting of all in-person learning opportunities in training platforms such as schools, hospitals and other clinical sites (Kumar et al., 2021), concerns were raised regarding appropriate clinical practice and even whether this could be successfully performed using online delivery. Therefore, the proposed move to online teaching was daunting, as most academic and clinical educators were unfamiliar with online teaching principles, theories and strategies (Al-Yateem et al., 2021). However, Khoza-Shangase, Moroe and Neille (2021) highlight the need for the South African speech–language pathology (SLP) profession to seriously consider this mode of delivery for the provision of clinical services, as well as for education and supervision even beyond the COVID-19 pandemic. For the current study, ‘education and training’ refers to teaching methods and activities for both theory and clinical modules in the undergraduate programme.

### Global, South African and institutional response to emergency remote online teaching and learning

Universities and schools across the world experienced lockdown and total closure of learning institutions around March 2020, impacting more than 94% of the world’s student population (Al-Yateem et al., 2021; Pokhrel & Chhetri, 2021). Thus, they had to discontinue face-to-face teaching to slow down the rise in numbers of new infections and deaths (Pokhrel & Chhetri, 2021). The decision paved the way for universities to introduce digital learning (Ferri, Grifoni, & Guzzo, 2020; Pokhrel & Chhetri, 2021), which raised both planning and implementation challenges for both students and educators (Oyedotun, 2020).

#### Challenges experienced by students

Psychological distress when working from home affected productivity (Pokhrel & Chhetri, 2021). This was compounded by factors such as taking care of sick family members, as well as worry about loss of income that paid for fees when parents lost jobs due to lockdown (Statistics South Africa, 2020). Other exacerbating factors to student engagement included mental health and challenges regarding domestic affairs. A Department of Higher Education and Training (DHET) survey conducted in 2020 on 13 000 students in South Africa reported that 40% of respondents could not buy food during the pandemic, whilst 30% did not have a suitable study space, 6% did not have electricity and more than 40% felt socially isolated (Krull & De Klerk, 2021). Whilst these realities are not new, emergency remote learning has brought them to the fore and required monitoring of students’ performance, health and participation by support and teaching staff (Al-Yateem et al., 2021; Oyedotun, 2020).

Stress factors such as minimal direction, especially for learners who usually struggled even in face-to face teaching and learning, and limited exposure to information and communication technologies (ICT) also exacerbated the struggle to access remote teaching and learning (Pokhrel & Chhetri, 2021). These challenges were particularly prevalent for students from disadvantaged backgrounds and communities, where even something as simple as connectivity was not guaranteed, and access not only to devices but also to data was limited (Pokhrel & Chhetri, 2021). In addition to these challenges in accessing learning resources, students in low- and middle-income countries (LMICs) such as Bhutan in South Asia had to work and assist on farms attending to cattle, doing home chores and other farming, and they often had no time for accessing learning in their home environments (Pokhrel & Chhetri, 2021).

In South Africa, several factors contributed to students’ capacity to move to remote learning, not least of which was the inequity of access to resources for students (Czerniewicz et al., 2020). At the university in which the study was conducted, most of the students come from disadvantaged backgrounds. This is in line with public universities’ policy to target the enrolment of students from disadvantaged communities (those in Quintile 1 and Quintile 2 schools) in South Africa (White & Van Dyk, 2019) to facilitate transformation and redress educational inequalities (Mestry & Ndhlovu, 2014). Quintile 1 schools in each province cater for the poorest 20% of learners, whilst Quintile 2 schools cater for the next poorest 20% of learners (Department of Education, 2004). A sizeable portion of students are therefore recipients of the National Student Financial Aid Scheme (NSFAS) by the South African government or other external sources of funding. Soon after the announcement of the national lockdown in 2020, the DHET communicated an adjusted budget for the provision of laptops for students funded by NSFAS (Songca, Ndebele, & Mbodila, 2021). However, there were delays in students receiving their laptops from NSFAS because of shipment challenges resulting from the national lockdown (Shoba, 2020). These students therefore had no laptops, which are a requirement for accessing resources and remote lectures and assessments. Furthermore, first-year students were doubly disadvantaged as their computer skills training did not happen during their orientation period as a result of student protests on campus and suspension of the academic programme at the beginning of the 2020 academic year, prior to the pandemic shutdown. Coronavirus disease 2019 in this case added an additional layer to historical disadvantage and unrest (Czerniewicz et al., 2020).

#### Challenges experienced by educators

Many educators required upskilling to be able to use ICT more effectively to transfer knowledge to their students. Thus, capacity development for remote teaching, learning and assessment (TLA) became an immediate focus for university leadership (Pokhrel & Chhetri, 2021). Educators felt ill-equipped to encourage student participation in an online environment where they were aware that students had limited use of ICT for the purpose of learning (Al-Yateem et al., 2021; Kumar, et al., 2021), and they were suddenly required to develop an online presence and engage with students (McGill, Turrietta, & Lal, 2021). The educators tended to describe students as unresponsive during online teaching and learning (Yakar, 2021). Common methods to facilitate engagement included online quizzes and interactive class sessions encouraging group work (Al-Yateem et al., 2021). Training for emergency remote teaching and learning (ERTL) for educators was sudden and covered copious amounts of content over a limited amount of time. Training was delivered online and required the use of multiple and often unfamiliar Internet-based programmes (Al-Yateem et al., 2021). In addition, digital inequalities, Internet inaccessibility, the lack of hardware such as laptops and the lack of adequate training for educators for online teaching were cited as resource constraints (Songca et al., 2021). There is conflicting evidence regarding the impact of emergency remote teaching (ERT) on learning. Al-Yateem et al. (2021) compared end-of-term results for 2019–2020 to those of 2020–2021 – in other words, for the period where teaching was all conducted face-to-face versus the period where online teaching and assessments was adopted. These researchers found higher scores for ERT than for face-to-face. On the other hand, Halilić and Tinjić (2020) found that scores for face-to-face assessments were higher than ERT assessments. However, Yakar (2021) found that despite the high scores achieved, educators agreed that the scores for ERT did not reflect the actual learning levels of students. These results should be interpreted with caution, as many variables could account for these differing findings. The reliability and quality of online assessments, cybersecurity issues leading to breaches and hacks, as well as a need for appropriate measures of checking for plagiarism are some of the concerns raised in the literature as possible influencing factors (Al-Yateem et al., 2021; Oyedotun, 2020; Pokhrel & Chhetri, 2021).

In addition, the onset of online teaching found educators taking on more technical, managerial and social roles. Educators encouraged students to develop an online learning community for peer support. Managing course designs, deadline extensions and creating engaging activities whilst remaining fair to both educators and students and maintaining academic integrity were seen as additional challenges that educators were required to navigate (Al-Yateem et al., 2021).

As a health science profession, it was important for SLP programmes to ensure the development of clinical skills through various online activities such as the use of videos and simulation software. The teaching of clinical skills in an online platform remained the most significant shift in the training of healthcare professionals (Al-Yateem et al., 2021).

### Transitioning to a different pedagogical approach

The higher education sector needed a strategy that would ensure reliable, fast, efficient and effective continuation of educational programmes. Thus began the unexpected shift to ‘ERT’ (Karakose, 2021). Emergency remote teaching is defined by Hodges and Fowler (2020, p. 119) as ‘a temporary shift of instructional delivery to an alternative delivery mode due to crisis circumstances’. For the purposes of the current study, ERTL has been used to include reference to teaching *and learning*, after Czerniewicz et al. (2020). Reference to ERTL includes both theory and clinical modules in the Bachelor of SLP programme. Much of the literature around ERTL focuses on theory, traditional lectures and tutorials. However, the practical aspects of the programme, including the clinical practicum, also had to be reimagined for ERTL.

Typically, developing an online course or module could take many months (Hodges & Fowler, 2020). Therefore, ERTL is not to be confused with online courses, which are specifically planned and designed to be delivered virtually (Hodges & Fowler, 2020; Mohmmed, Khidhir, Nazeer, & Vijayan, 2020; Petersen, n.d.). The COVID-19 pandemic circumstances required educators and students to transition quickly from face-to-face TLA to some form of ERTL. As a result, both educators and students were underprepared (Karakose, 2021; Jelinska & Paradowski, 2021). Upskilling needed to be fast (Mohmmed et al., 2021), and institutions rolled out online courses, webinars and self-study materials for educators and students to support this upskilling, and this all happened simultaneously to the actual TLA.

The reference to the quick transition should not imply poor pedagogy or inferior quality. It is imperative to ensure that even though delivery methods may change, the quality of the learning outcomes is not compromised, and outcomes are still met (Hodges & Fowler, 2020; Petersen, n.d.). Although the focus of the current study is on the ERTL as a response to the COVID-19 pandemic at the university, it is likely that online learning in some form or another is here to stay (Jelinska & Paradowski, 2021; Karakose, 2021). Lessons learned from current experiences can therefore inform curriculum development for the future.

### Opportunities created by emergency remote teaching and learning

Despite the significant challenges experienced in the transition to ERTL, literature also recognised opportunities that the pandemic created. Pokhrel and Chhetri (2021) highlighted the following opportunities: building strong connections whilst navigating through ERTL, as well as exploration of the use of online platforms (e.g. Google Classroom and Zoom) and social media group forums (e.g. WhatsApp and Messenger) as additional resources to strengthen teaching and learning programmes. There was also opportunity for increased collaboration between educators and students in finding creative solutions to overcome the restrictions of ERTL (Pokhrel & Chhetri, 2021). Karakose (2021) identified the prospects of reassessing and restructuring higher education systems from a reformist perspective to combine technology-supported digital learning together with traditional face-to-face in a hybrid model to facilitate TLA, particularly for socially disadvantaged students who may find the cost of attending university face-to-face prohibitive. Online opportunities could also be explored to increase digital literacy, provide psychological support for both educators and students and identify fun online strategies to increase student motivation (Karakose, 2021).

The review literature has presented the global, South African and institutional response to ERTL with particular focus on the challenges experienced by students and educators whilst also acknowledging the opportunities created by ERTL. The relevant literature substantiates the importance of the current study, which aimed to explore the SLP educators’ experiences of the planning and implementation of ERTL during the COVID-19 pandemic in a South African university.

## Methodology

### Research question

How did SLP educators experience the planning and implementation of ERTL during the COVID-19 pandemic in a South African university?

### Aim

To explore the educators’ experiences of the planning and implementation of ERTL during the COVID-19 pandemic in a South African university.

### Objectives

1. To explore the educators’ experiences of the planning of ERTL during the COVID-19 pandemic.
2. To explore the educators’ experiences of the implementation of ERTL during the COVID-19 pandemic.

### Study design

A qualitative, descriptive narrative design (Edmonds & Kennedy, 2017) was used to explore the experiences of educators who engaged in ERTL during the COVID-19 pandemic. This approach was most suitable as it allowed for the unravelling of ‘consequential stories of people’s lives as told by them in their own words and worlds’ (Ntinda, 2018, p. 1). A narrative design presented an opportunity for educators to document and reflect on their practice to support, evaluate and improve teaching and learning.

### Setting

The study was conducted at a university in South Africa that offers education and training in SLP.

### Study population and sampling strategy

A purposive nonprobability sampling technique was utilised to recruit full-time, permanent educators in the discipline of SLP. An invitation was e-mailed to all potential educators who met the inclusion criteria, and seven agreed to participate in the study. The inclusion criteria required participants to be SLP educators and to have been involved in the planning and implementation of education and training in the undergraduate programme through ERTL during the COVID-19 pandemic in 2020.

### Procedures and data collection

**FIGURE 1:**
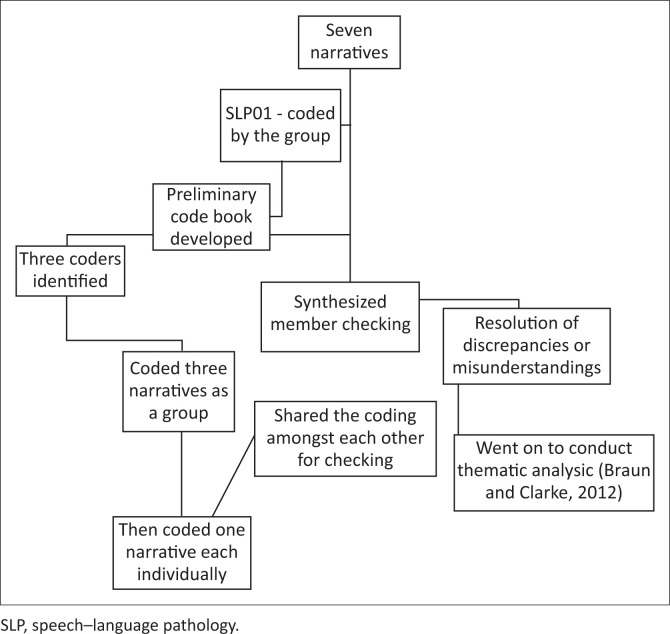
Process followed in coding the narratives.

The invitation that was emailed outlined the study title, aim and objectives. Educators were required to use the research objectives as prompts to reflect on their experiences and produce a reflective narrative. All narratives were submitted in English, as all participants were proficient in English. Submissions were e-mailed to the research coordinator (i.e., first author in this article who is also an educator). The narratives were coded by the research coordinator in the first level of analysis to ensure anonymity and confidentiality. Once the narratives were received and coded, they were stored in a password-protected file on Microsoft (MS) Teams. The file was accessible to the seven members of the research team for data analysis. The coding process is described in Figure 1.

### Data analysis

An inductive thematic analysis of the narratives was guided by Braun and Clarke’s (2012) six-step process. In the first step, six researchers familiarised themselves with one narrative and developed the preliminary codebook. All codes were named by consensus in second step. The balance of the narratives was then coded by a smaller cohort of three researchers, and the codebook was expanded. The coded transcripts were shared with all participants for member checking to ensure credibility of the data. In the third step, themes were identified by consensus in a group meeting. Themes were represented diagrammatically and circulated for review (fourth step) and naming, which was again done by consensus (fifth step). The report was then finalised (sixth step).

### Trustworthiness

Guba (1981) proposed four criteria to ensure trustworthiness in a qualitative study. (1) Credibility was ensured by carrying out member checking. (2) Transferability was ensured by detailing all the procedures. (3) Confirmability was ensured by involving three coders during data analysis. (4) Dependability was ensured in this study by describing the procedures undertaken for study (i.e. data collection and data analysis). An audit trail was ensured by storing all the data and notes for data collection and analysis in password-protected documents.

### Ethical considerations

Ethical clearance was obtained from the university’s Research Ethics Committee (reference number: HSSREC/00003784/2022). All ethical considerations such as informed consent, confidentiality, beneficence and non-maleficence were adhered to when this study was conducted. Additionally, the raw data is kept in password-protected Microsoft Word 2019 documents which only the research team has access to.

## Findings

Seven participants were included in the study. Participants were all female, representing a diverse cultural profile, with clinical and/or educator experience ranging from less than 5 years to over 20 years, within the age range of 35–65 years.

During the process of data analysis, it became clear that presenting findings for objectives separately would potentially compromise the richness of the data. The iterative nature of the process made it difficult to differentiate experiences of planning and implementation. Thus, the themes presented below therefore include both phases, and the findings are presented as a composite of objectives 1 and 2.

**FIGURE 2:**
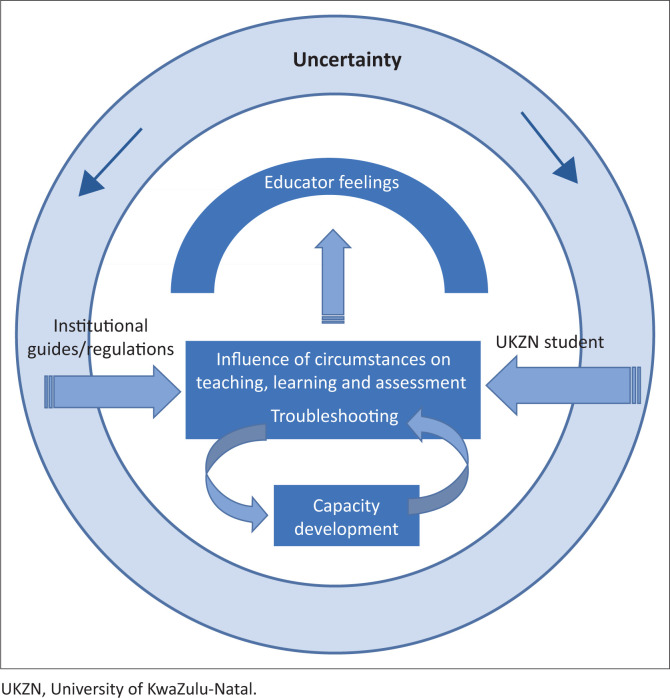
Themes dominant in the study.

Five dominant themes emerged from the data gathered and are illustrated in Figure 2. The five themes included uncertainty, educator feelings, capacity development, influence of circumstances on TLA and troubleshooting. Figure 2 further illustrates the relationship amongst the themes. Throughout the planning and implementation process, the adaptations to and reimagining of the TLA were shaped by multiple influences. These included institutional influences such as guidelines, the need to cater to the uniqueness of the university student, as well as educators’ awareness of and sensitivity to the students’ needs, all of which were impacted on and by the educators’ feelings and emotions. Capacity development was ongoing and iterative, as educators made amendments to the programme and continued to develop new skills. Through this process, educators grew in confidence and competence but have not yet reached a point of certainty.

### Theme 1: Uncertainty (context)

Uncertainty is presented and discussed as the backdrop or context in which all other themes are embedded. This was an unprecedented time, globally, in which educators found themselves. There was a lack of clarity around the COVID-19 disease progression, prevention and its effects on all sectors, including education. The global move to ERTL was an inevitable consequence and a change from familiar pedagogies. The only ‘certainty’ was that change was necessary and inevitable. Uncertainty permeated through all sectors, including education. Information poured in from various media, some credible, some not. Confusion reigned. Decisions were being made by scientists, politicians and legal experts that would impact the lives of millions, without a clear idea of how the pandemic would play out:

‘We were so uncertain, you know. We were hoping maybe by a certain date or after the initial hard lockdown that students would return. At the time, I think the uncertainty of not even knowing how to treat COVID-19, which contributed to the reluctance or caution on the side of the university…’ (SLP07)

‘As we started focusing on the academic programme, it was a feeling of, “where do we start in continuing with the programme?” Students were now at home (including those who lived at university residences). Initially, departments were left to figure things out on their own – which was very stressful. Communication from the school and university leadership was initially slow – whilst they themselves were trying to plan.’ (SLP03)

The decisions about ensuring continuity of education at a national or institutional level provided the initial impetus regarding programme adaptation for ERTL. However, there was a sense of confusion as information from various sources was contradictory, and guidance on clinical training was absent, with institutional guidance being slow. There was uncertainty about physical student contact and resumption of the programme, even if online. It was even uncertain whether all students had access to devices that would enable the educators to use ERTL:

‘The discussion amongst staff centred around what it would be like, are we really in a pandemic, what would happen in South Africa, how would we teach? There was so much uncertainty of what was going to happen. … [*The students*] were frantic that they had to take what they could and leave; some were worried about not having their own laptops and how they were going to cope. They wanted to know plans, and at that stage, we had not received specific plans regarding teaching and learning from management.’ (SLP07)

### Theme 2: Educator feelings

A feeling of being anxious and overwhelmed at the onset of the pandemic immediately followed student protest action at the university in March 2020. The focus at the start of the pandemic was not on the academic programme but on learning about the COVID-19 virus, its global impact and managing a household that was in lockdown.

The focus on educator training to upskill and migrate to an online training platform within a short space of time left educators feeling exhausted, with some feeling ambushed by the magnitude of the content covered during training:

‘Development of online clinical training activities as well as online activities for theory modules can be quite time consuming and overwhelming for staff, as it requires a shift in teaching pedagogy, creativity and ensuring that you are presenting content to cater for various students.’ (SLP 05)

Other educators were not previously ‘tech savvy’, and the sudden move to ERTL left them uncomfortable. Feelings of discomfort were lessened by learning from colleagues and listening to their experiences. Planning of modules, especially clinical modules, left educators feeling anxious and stressed as clinical sites closed their doors to student training, prioritising the management of patients with COVID-19:

‘But what about academic staff? If no student is to be left behind, then surely someone must lead? Was that supposed to be us? Me? Those feelings of being overwhelmed returned.’ (SLP 02)

Exacerbating the feelings of anxiety was educator awareness that students were experiencing their own stressors, including managing domestic, technological, infrastructure and load-shedding challenges. Emergency remote teaching and learning left educators feeling isolated and disconnected from their students and colleagues, with limited human contact whilst dealing with ERTL challenges:

‘I did not have human contact – the sense of community from my peers and students.’ (SLP 02)

The laptop screen was viewed as both a physical and social barrier. Whilst an educator support network was available in the form of staff meetings, due to data and connectivity challenges, laptop mics were often muted and cameras switched off, making the experience a faceless exchange.

Despite these negative feelings, many educators were excited to reimagine their teaching and were eager to learn. Educator confidence improved as the ideas and resources to develop online teaching activities were shared with them.

### Theme 3: Capacity development

This theme refers to educators undertaking in-service training and self-directed learning to upskill for the shift to ERTL. Institutional audits supported the need for capacity development of educators. In this theme, educators’ experiences of methods to enhance digital literacy for ERTL are explored.

At the outset, educators realised that their current understanding and implementation of blended learning was limited, and they lacked the requisite competence for an immediate shift to ERTL:

‘[…*U*]niversity had an online teaching platform, but we used it … scarcely … more a repository for documents and quizzes prior to 2020. Our skills in using the online teaching platform were not excellent … it was not something we were dependent on with face-to-face lectures. Our previous teaching was very much a traditional approach. … We had to ensure that we had the technical skills to now transfer our teaching onto the online platform.’ (SLP07)

‘Staff were required to not only learn about and establish for themselves the online teaching pedagogy for their modules but also to navigate the various platforms such as Moodle, Zoom and Simucase that we would use to deliver training and assessments.’ (SLP05)

Although educators did benefit from in-service training, there were challenges. Training demands increased rapidly, but the training did not follow a meaningful, developmental format that would have facilitated educators’ understanding. Educators felt that the training focused more on knowledge rather than skills:

‘There was too much training going on from the university. They were trying to overload us with knowledge without skills in a short space of time. I feel that everything was a mess. …’ (SLP06)

Whilst educators were trained to transition to ERTL for theory modules, a similar focus for online clinical intervention methods such as telerehabilitation was absent. This was a significant challenge for educators in the department, because these intervention methods had not been previously routinely utilised, and therefore curriculum focus was limited:

‘I have never done telerehab ever before in my practice, and now I must speak from a point of authority on the subject. This is where the added pressure comes in. As a profession[*al*], I have not come across courses or webinars addressing this issue. I just listen to others or read [*on social media*] what others are doing. It is very much trial and error.’ (SLP02)

### Theme 4: Influence of circumstances on teaching, learning and assessment

This theme refers to how circumstances guided decisions regarding adaptations of the curriculum for ERTL. In this theme, the researchers present how the designing of TLA methods and strategies was informed by awareness of and sensitivity to the student challenges within this context. The university’s focus on redressing inequities of the past by allocating a greater number of first-entry admissions to students from Quintile 1 and 2 schools. This implies that most students come from significantly disadvantaged backgrounds.

The educators reflected on the student circumstances and challenges that informed their TLA. This included data affordability, poor infrastructure affecting connectivity, access to water and power, limited access to devices that may be used for learning and assessment, learning environments that may not be conducive and the need to participate in household chores:

‘The socio-economic backgrounds our students came from, the geographical areas that the students would have gone back to was to think about access and where the students were. From those perspectives, we tried to conduct a quick online survey to see where students were, what devices they had and whether they had access to the Internet.’ (SLP07)

Coronavirus disease 2019 itself was a challenge, as students were either infected or affected in some way by the virus. Groupwork during this time was challenging, as students were unable to find common times to work and when they did, connectivity was unstable, impacting on completion time. Lower data costs meant students preferred working at night. Students in their first year of study were under-prepared for learning, as they had only arrived at university before its closure:

‘Because we were so aware of data costs, access, nature of devices students had access to and living conditions, very little contact was made with videos on, particularly for students.’ (SLP 01)

Decisions about TLA were guided by the different regulatory bodies such as the Health Professions Council of South Africa (HPCSA) and the University Teaching and Learning Committee. Decisions included the transition to ERTL, the use of various online methodologies for clinical training and a flipped classroom–hybrid model of TLA. Lecture content was modified to include easily accessible and downloadable texts to all students, most of whom were using their smartphones with limited data. The reading material for the flipped classroom approach needed to be user-friendly, as most students are second-language English learners who needed to engage with the content in their own time, with pertinent issues discussed during ‘live’ synchronous lectures:

‘[…*R*]eading material that was going to be user-friendly, accessible online, downloadable even on cell phones, so that students didn’t waste data.’ (SLP07)

‘The students reflected before the lesson and made the lesson easier to facilitate as they had prior knowledge.’ (SLP06)

The migration to ERTL meant that teaching methods needed to be adapted. Planning throughout was necessary for theory and clinical modules. For clinical practice, simulated cases, paper-based cases and videos were developed for students. As most venues closed their doors and others had restricted access to clients, clinic planning from an administrative focus took much effort:

‘Planning therefore developed from the areas that I felt could be covered well by simulated cases, within the limits of the hours allowed, and then I explored other ways of meeting remaining outcomes…’ (SLP01)

The directive from the university was that ‘live’ synchronous teaching for theory modules needed to be reduced, allowing students to engage with learning materials and lectures in a time that was convenient for them, considering data costs and infrastructure issues. Educators prerecorded lectures, did voiceover PowerPoints, uploaded readings and selected appropriate videos for students. However, a challenge was that student engagement was reduced during lectures:

‘Students were mostly unresponsive, and it was so easy to go back to our familiar way of lecturing during live Zoom or send prerecorded slides.’ (SLP04)

An ongoing concern was whether students were meeting module outcomes. The lack of active student engagement during lectures made this difficult to discern. One way in which this was managed was through ongoing assessment in modules, whether developmental or formative.

Clinical practice has traditionally followed a continuous assessment format. This did not change, but the activities for assessment were varied to include simulations, role play, case studies and telerehabilitation sessions (synchronous and asynchronous):

‘[…*S*]tudents were provided with real-life clinical scenarios whilst also ensuring that all module outcomes (assessment, management, screening, prevention, promotion, counselling and education) were met within the different online clinical activities allowed, i.e. simulations, case studies, role play and telerehab.’ (SLP05)

A generic fourth-year module was also developed to facilitate consolidation of skills:

‘[…*S*]taff prioritised fourth-year and used the time to analyse and identify gaps in knowledge and skills – which could be addressed though online teaching….’ (SLP03)

Despite all the adaptations, educators still felt that assessment remained problematic:

‘We have yet to learn ways of creating online assessments that ensure that students do not copy. We have trialled some changes with time, shuffling questions, etc., but still need other ways to ensure results are not compromised. The students’ final marks were extremely high[*er*] than average; this can be attributed to weak systems that can stop plagiarism and copying.’ (SLP04)

### Theme 5: Troubleshooting

It was clear that challenges occurred before the planning phase was started, and many more arose thereafter. At times these overwhelmed both educators and students. However every effort was made to ‘troubleshoot’ as each situation arose. The theme ‘troubleshooting’ therefore refers to attempts to address challenges experienced by educators and students during ERTL and solutions.

Communication was of particular concern as students returned to their homes, often in rural and remote areas. The exiting (fourth-level) students were prioritised to ensure they met the requisite competencies within the (disrupted) academic year. Student support was therefore key, and efficient communication was necessary. However, concerns about connectivity and data costs resulted in quick, simple and cost-effective communication methods. Most educators and students found WhatsApp the most reliable solution. It was also used to support students who were unable to log into Zoom sessions, by using speaker-phone to allow full participation:

‘Communication was difficult. WhatsApp was used to set up chat groups with class reps. This was used to ascertain information about the class – regarding who had access to e-mail [*and*] WhatsApp and who didn’t. Eventually chats groups were created for modules with those students who had access, BUT there were many who didn’t – since many students at the university came from socio-economically disadvantaged backgrounds (selected from Quintile 1–3 schools) – university’s admissions policy addressing inequities in the majority students’ access to university.’ (SLP03)

‘WhatsApp access meant I was often contacted well out of office hours, over weekends or late at night, and despite the temptation to wait until the appropriate time to read the messages, I tried to be sensitive to students’ need for reassurance or to deal with queries quickly.’ (SLP01)

The second quote indicates how at times the ‘solutions’ in themselves potentially became problems.

## Discussion

The findings of this study provided insight to the challenges encountered and strategies utilised by educators in planning and implementing ERTL. Although ERTL was introduced at the university, which was already using hybrid models of training, it was still daunting for educators because of limited knowledge and skills of ERTL, which led to uncertainty. The use of learning management systems (LMSs) in TLA requires both computer and digital literacy (Mohammadyari & Singh, 2015). Limited digital literacy might have resulted in apprehensiveness, because adapting and accepting recent technology can be challenging. It was crucial that educators attended training to enhance their knowledge and skills to effectively use the LMS and other online tools for teaching theory and clinical modules. Whilst this training was useful, it lacked theoretical coherence and focused more on knowledge than skills. This prevented immediate application, thereby exacerbating the uncertainty.

The emotional response by educators within the department of SLP is indicative of the uncertain circumstances around the delivery of ERTL within a pandemic and the challenges of the university context. Whilst mixed feelings were a common thread through the initial planning and implementation phases, educators developed confidence and a sense of security as they resolved the challenges arising from ERTL and supported each other. These sentiments are similar to the findings in a study by Meishar-Tal and Levenberg (2021), which indicated that lecturers experienced ‘threat’ and ‘failure’ more intensely at the start of ERTL, as they lacked any formal training or competency in this method of delivery. Their anxiety was heightened due to having to provide ERTL during the pandemic. However, as their competency in ERTL increased, more positive emotions of success were evoked.

Educators collaborated around evidence-based solutions to facilitate clinical training. Clinical competencies were met by including simulations, telerehabilitation and case studies. These strategies were used to supplement clinical training due to limited access to sites. Educators and students engaged in collaborative teamwork to solve problems, forging ahead to save the academic programme. Subsequently, the department had to report back to other bodies for approval of these strategies.

Low-tech communication tools such as WhatsApp and e-mail were mostly used between educators and students, similar to the findings in studies by Alawamleh et al. (2021), Al-Yateem et al. (2021) and Oyedotun (2020). Teaching platforms like Zoom and Moodle (the learning management system [LMS] used at the university) were used in the current study.

Alawamleh et al. (2021) reported students feeling disconnected from educators and classmates, whereas in the current study educators felt that these platforms, particularly WhatsApp, helped with human contact. Collaboration and communication occurred amongst educators and students within and amongst institutions, which helped navigate through the uncertainty. Similar findings were reported at other universities nationally and internationally (Czerniewicz et al., 2020; Pokhrel & Chhetri, 2021). In this study, social media and technology that were easily available were used initially (i.e. WhatsApp, e-mail) with other methods incorporated later as knowledge and skills increased (e.g. Zoom).

The perceived circumstances and challenges encountered by SLP students at the university were not different from those of students from other LMICs (Oyedotun, 2020). Issues were related to data affordability, poor connectivity, lack of devices and limited time for learning due to domestic chores (Matsilele, 2021; Pokhrel & Chhetri, 2021). This resulted in students opting to complete assessments and catch up on recorded lecture content at night when data costs were lower and/or chores were done (Pokhrel & Chhetri, 2021), indicating the resilience and perseverance of students.

Challenges were common across students, which was more evident with first-year students due to the lack of timely institutional support resulting from protest action at the university in the weeks preceding lockdown. The inequalities that existed before COVID-19 were further emphasised by ERLT (Czerniewicz et al., 2020; Krull & De Klerk, 2021). Educator sensitivity to students from lower socio-economic backgrounds when planning and implementing the move to ERTL was required (Al-Yateem et al., 2021; Pokhrel & Chhetri, 2021). The results highlighted educator challenges in ensuring that ERTL was student-centred so that no student was left behind. This made planning and implementing ERTL difficult. Whilst the institution adopted a student-centred approach from the outset, educators struggled to cope with the additional demands that came with student support and capacity development. It was important for the institution to meet the needs of both educators and students simultaneously. Karakose (2021) stresses the need for educators to receive relevant psychosocial support as well.

## Conclusion and recommendations

The university’s imperative that no student be left behind illuminated the inequities in the student body. Educators could not make assumptions that students had access to devices and conducive environments to support ERTL. Decisions about TLA had to be made with sensitivity to and an awareness of student circumstances. Whilst educators acknowledged several challenges, feeling overwhelmed, stressed and anxious, these feelings dissipated to some extent as they became more skilled and therefore comfortable in an online environment. It is recommended that the ERTL response for a time of crisis should be designed beforehand from a preparedness approach rather than a reactionary one, which is what happened during the start of the COVID-19 pandemic. Organisational communication in times of crisis should also be planned and implemented effectively to reduce feelings of uncertainty and anxiety. The opportunity for educators to enhance their digital literacy means that improved confidence and competence will result in a hybrid model and continued use of online TLA, regardless of the return to face-to-face teaching. Online platforms have also facilitated more collaboration between students, between students and educators and between educators within the department and internationally. There is a need for building educators’ capacity to navigate the learning management systems more efficiently, especially as technologies evolve. Furthermore, opportunities to support students through social media platforms could result in students receiving more immediate and individual support.
